# Overlapping Roles of Yeast Transporters Aqr1, Qdr2, and Qdr3 in Amino Acid Excretion and Cross-Feeding of Lactic Acid Bacteria

**DOI:** 10.3389/fmicb.2021.752742

**Published:** 2021-11-23

**Authors:** George C. Kapetanakis, Christos Gournas, Martine Prévost, Isabelle Georis, Bruno André

**Affiliations:** ^1^Molecular Physiology of the Cell, Université Libre de Bruxelles, Biopark, Gosselies, Belgium; ^2^Microbial Molecular Genetics Laboratory, Institute of Biosciences and Applications, National Centre for Scientific Research “Demokritos”, Agia Paraskevi, Greece; ^3^Structure et Fonction des Membranes Biologiques, Université Libre de Bruxelles, Brussels, Belgium; ^4^Transport of Amino Acids, Sensing and Signaling in Eukaryotes, Labiris, Brussels, Belgium

**Keywords:** *Saccharomyces cerevisiae*, yeast, lactic acid bacteria, amino acid transport, cross-feeding, *Lactobacillus fermentum*, amino acid excretion, drug H^+^ antiporter

## Abstract

Microbial species occupying the same ecological niche or codeveloping during a fermentation process can exchange metabolites and mutualistically influence each other’s metabolic states. For instance, yeast can excrete amino acids, thereby cross-feeding lactic acid bacteria unable to grow without an external amino acid supply. The yeast membrane transporters involved in amino acid excretion remain poorly known. Using a yeast mutant overproducing and excreting threonine (Thr) and its precursor homoserine (Hom), we show that excretion of both amino acids involves the Aqr1, Qdr2, and Qdr3 proteins of the Drug H^+^-Antiporter Family (DHA1) family. We further investigated Aqr1 as a representative of these closely related amino acid exporters. In particular, structural modeling and molecular docking coupled to mutagenesis experiments and excretion assays enabled us to identify residues in the Aqr1 substrate-binding pocket that are crucial for Thr and/or Hom export. We then co-cultivated yeast and *Lactobacillus fermentum* in an amino-acid-free medium and found a yeast mutant lacking Aqr1, Qdr2, and Qdr3 to display a reduced ability to sustain the growth of this lactic acid bacterium, a phenotype not observed with strains lacking only one of these transporters. This study highlights the importance of yeast DHA1 transporters in amino acid excretion and mutualistic interaction with lactic acid bacteria.

## Introduction

Amino acids are among the most abundant nitrogenous compounds in all cells. They serve a wide range of purposes, as building blocks for protein synthesis, as nitrogen or carbon sources, as metabolic intermediates, and as chemical messengers (Gutiérrez-Preciado et al., 2010). Their uptake across the plasma membrane typically involves transporters that have been extensively investigated for decades in many species. For instance, the yeast *Saccharomyces cerevisiae* possesses about 20 plasma-membrane amino acid transporters. Most of these proteins belong to the highly diverse Amino acid-Polyamine-organoCation (APC) transporter superfamily and are subject to well-documented multiple-level regulations (Regenberg et al., 1999; Jack et al., 2000; Van Belle and André, 2001; Bianchi et al., 2019). Under particular conditions, *S. cerevisiae* can also excrete amino acids. For instance, a pioneering study by S. W. Challinor and A. H. Rose showed that yeast cells, when faced with a suboptimal nutrient supply, excrete several compounds. They notably excrete amino acids in sufficient quantity to meet the amino acid requirements of co-cultivated lactic acid bacteria (Challinor and Rose, 1954). Twenty years after this study, M. Grenson reported that amino acid excretion by yeast is favored in situations of metabolic imbalance such as partial auxotrophy, impaired nutrient utilization, or growth arrest due to starvation for a specific nutrient (Grenson, 1973). In the same study it was shown that yeast cells lacking arginine permease activity excrete arginine, as shown by their ability to cross-feed an arginine auxotroph. This indicated that amino acid importers can potentially mediate retention of specific amino acids that naturally leak out of the cell (Grenson, 1973). Subsequent studies have shown that yeast excretes detectable levels of amino acids when they are overproduced (Velasco et al., 2004) and when ammonium uptake and its assimilation into amino acids are abnormally high (Hess et al., 2006). Amino acids have also been detected in the exometabolome of yeast grown under conditions favoring overflow metabolism (Paczia et al., 2012). A more recent study has similarly demonstrated that metabolically active yeast cells growing on a nitrogen-rich medium excrete metabolites, especially amino acids, and that this excretion enables co-cultivated lactic acid bacteria to proliferate (Ponomarova et al., 2017). Furthermore, on a lactose medium these bacteria release lactose-derived galactose and glucose, which can be used as carbon sources by yeast cells. This highlights actual mutualism between the two co-cultivated microorganisms (Ponomarova et al., 2017). More generally, it is established that microorganisms occupying the same ecological niche or codeveloping during food product fermentation can exchange metabolites, notably amino acids, and mutualistically influence each other’s metabolic differentiation (Martin-Rendon et al., 1993; Scherlach et al., 2013; D’Souza et al., 2018; Tshikantwa et al., 2018).

Although amino acid export by yeast cells has been reported for many years, the transport systems involved remain poorly known. In a previous study, we described a yeast strain excreting threonine (Thr) and its precursor homoserine (Hom). Two conditions had to be fulfilled for this excretion to reach readily detectable levels. First, both amino acids had to be overproduced by the cells, which was achieved by expressing a mutant aspartate kinase (involved in Hom and Thr biogenesis) resistant to feedback inhibition (*HOM3-R*) (Martin-Rendon et al., 1993). Second, the three main Thr and Hom plasma-membrane permeases, Gap1, Agp1, and Gnp1, had to be inactivated to reduce reuptake of both excreted amino acids. We also found the nitrogen source of the medium to have a strong influence on Thr excretion at least. Under the conditions just mentioned, Thr and Hom were excreted into the external medium to levels allowing detectable cross-feeding (CF), on solid medium, of Thr- and Hom-auxotrophic yeast mutants. Further experiments revealed an important role of Aqr1, a protein of the Drug H^+^-Antiporter 1 (DHA1) family, in Hom excretion (Velasco et al., 2004). The DHA1 family belongs to the Major Facilitator Superfamily (MFS) (Sá-Correia and Tenreiro, 2002; Tenreiro et al., 2002), the largest known superfamily of carriers, represented in all kingdoms of life (Reddy et al., 2012). Among the 12 yeast DHA1-family proteins, many, including Aqr1, are reported to confer resistance to drugs (Sá-Correia et al., 2009). Some of these are involved in metabolite excretion, however, suggesting that DHA1 members capable of conferring multidrug resistance might fortuitously or opportunistically recognize toxic compounds (drugs) but be mainly involved in excretion of metabolites, including amino acids (Sá-Correia et al., 2009). In support of this view, among the yeast DHA1 members, the Dtr1/Spo14 transporter mediates excretion of bisformyl-tyrosine, a natural ascospore cell component (Felder et al., 2002), and the Tpo1-4 proteins are reported to transport polyamines (Felder et al., 2002). Furthermore, transcription of the closely related genes *AQR1* and *QDR1-3* is regulated by nitrogen or amino acid availability (Velasco et al., 2004; Sá-Correia et al., 2009; Dos Santos et al., 2014), and a lack of Qdr2 alters amino acid homeostasis (Vargas et al., 2007).

In this study we have investigated further the role of DHA1-family transporters in amino acid excretion and their potential contribution to cross-feeding lactic acid bacteria. We show that a lack of Aqr1 and Qdr3 results in loss of Hom excretion and that Thr excretion is prevented when a third DHA1 protein, Qdr2, is also inactive. We report differences between these three proteins as regards subcellular location and intracellular trafficking. We have built and exploited a structural model of the Aqr1 exporter to identify residues in its substrate-binding pocket that play an important role in amino acid excretion. Finally, we report that a mutant strain devoid of Aqr1, Qdr2, and Qdr3 displays a reduced ability to cross-feed a lactic acid bacterium. Our study sheds new light on the molecular mechanisms of amino acid excretion by yeast and on their importance in symbiotic interactions with lactic acid bacteria.

## Materials and Methods

### Strains and Growth Conditions

All the *Saccharomyces cerevisiae* strains used in this study (Supplementary Table 2) derive from the Σ1278b wild type (Bechet et al., 1970). The plasmids used are listed in Supplementary Table 3. Unless otherwise stated, yeast cells were grown at 29°C in minimal citrate-buffered (pH 6.1) medium (code numbers 165 for liquid and 167 for solid media) (Jacobs et al., 1980), with 3% glucose as the carbon source and 0.1% L-glutamic acid as the nitrogen source. In experiments where gene expression was driven by the *GAL1-10* promoter, cells were grown overnight in minimal medium containing 3% galactose and 0.3% glucose as carbon sources and then shifted to 3% glucose 1.5 h before analysis.

Yeast and *Lactobacillus fermentum* (Beijerinck 1901 AL) were co-cultivated on a minimal medium containing glucose (3%) as a carbon source and ammonium as a nitrogen source added as (NH_4_)_2_SO_4_ (0.5%). This medium (code number 169) is equivalent to 165 (Jacobs et al., 1980) except that citrate is replaced with MES hydrate (19.5 g.L^–1^) as a buffer (pH 6.1) and that additional compounds are provided: folic acid (0.056 mg.ml^–1^), nicotinic acid (0.09 mg.ml^–1^), 4-aminobenzoic acid (0.0056 mg.ml^–1^), riboflavin (0.09 mg.ml^–1^), reduced L-glutathione (1.5 mg.ml^–1^), and cobalt(II) chloride (0.19 mg.ml^–1^). Cultures of *L. fermentum* alone were also supplied with amino acids (yeast synthetic drop-out without histidine, Sigma-Aldrich). For co-cultures, yeast and *L. fermentum* were first cultivated separately in 169 medium, collected by centrifugation, and washed twice in 169 medium. Cell suspension samples of equivalent optical density (∼0.05 at 660 nm) were used to fill 6-well plates equipped with ThinCert cell culture inserts (GREINER-657640, as outlined in Figure 5B). Each well was first filled with 2.5 ml yeast cell suspension and the Thincert insert containing 2 ml *L. fermentum* cell suspension was placed on top of it. The co-cultures were incubated for 48 h at 29°C with shaking at 100 rpm. Samples collected just after and 48 h after inoculation were diluted for counting the colony-forming units (CFUs) on rich medium.

**FIGURE 1:**
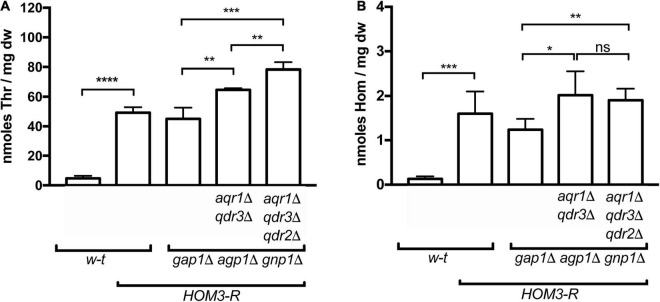
Overproduction of threonine and homoserine in yeast strains used in cross-feeding assays. Intracellular concentrations of threonine (Thr) **(A)** and homoserine (Hom) **(B)** were quantified by UPLC in wild-type and mutant strains (genotypes indicated), transformed with plasmid pGK007 (*HOM3-R*) or the empty vector. Cells were grown in a minimal glucose medium with glutamate as sole nitrogen source. Bars represent averages of four independent experiments ± standard deviation. * indicates a statistically significant difference as determined with the unpaired *t-*test. *P < 0.0332; **P < 0.0021; ***P < 0.0002; and ****P < 0.0001. ns, not significant, *P* > 0.05.

**FIGURE 2:**
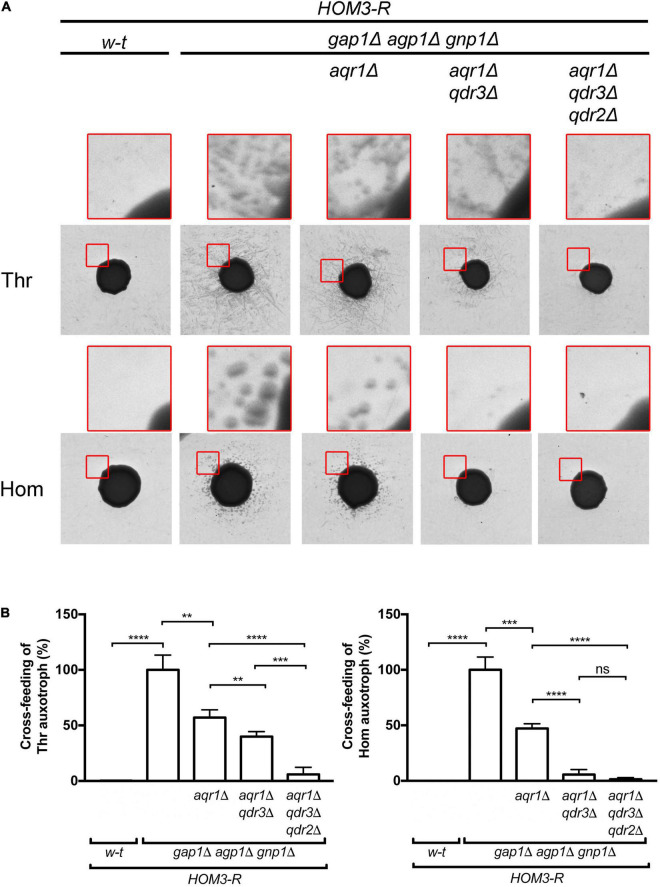
Aqr1, Qdr2, and Qdr3 contribute to threonine and homoserine excretion. **(A)** Cross-feeding on solid medium of threonine (Thr) and homoserine (Hom) yeast auxotrophic mutants surrounding large colonies of the wild-type (*w-t*) or a mutant strain (genotypes indicated). All donor strains expressed *HOM3-R* from a plasmid (pGK008). Cells were grown in a minimal glucose medium with glutamate as sole nitrogen source. The presented images correspond to the inverted grayscale of original scans obtained for a representative experiment (original images are available in Supplementary Figure 1). Upper images in red squares correspond to enlargements of a zone at the periphery of the central donor colony. **(B)** Cross-feeding results as illustrated in A were quantified by image digitization, measurement of the pixel values corresponding to satellite colonies of the auxotrophic strains, and normalization vs. the strain with functional *AQR1*, *QDR2*, and *QDR3* genes. Bars represent averages of four independent experiments ± standard deviation. * indicates a statistically significant difference as determined with the unpaired *t-*test. **P < 0.0021; ***P < 0.0002; and ****P < 0.0001. ns, not significant, *P* > 0.05.

**FIGURE 3:**
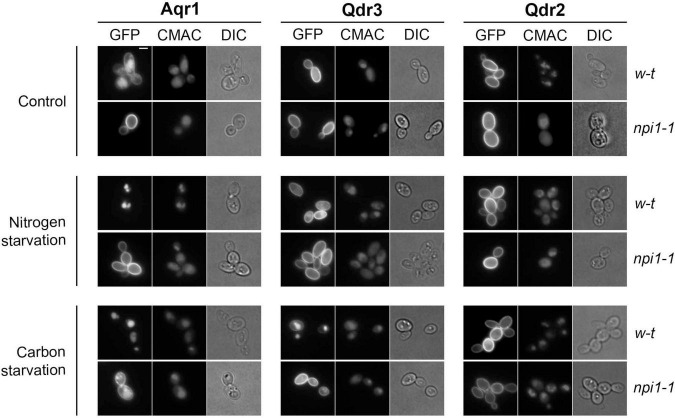
Aqr1, Qdr2, and Qdr3 show distinct subcellular localization patterns under normal and starvation conditions. Cells of the wild-type strain and *npi1-1* endocytosis mutant, expressing the hybrid gene *AQR1-GFP, QDR3-GFP*, or *QDR2-GFP* at the natural gene’s chromosomal locus from the *GAL1-10* promoter, were initially grown overnight in minimal medium containing galactose (3%), glucose (0.3%), and glutamate. They were then collected, washed, and transferred to the same medium containing glucose (3%) and glutamate (Control), glucose (3%) but no glutamate (nitrogen starvation), or glutamate but no glucose (carbon starvation). After 90 min, the cells were observed by epifluorescence microscopy. CMAC dye was used to label the vacuolar lumen. Scale bar = 2 μm.

**FIGURE 4:**
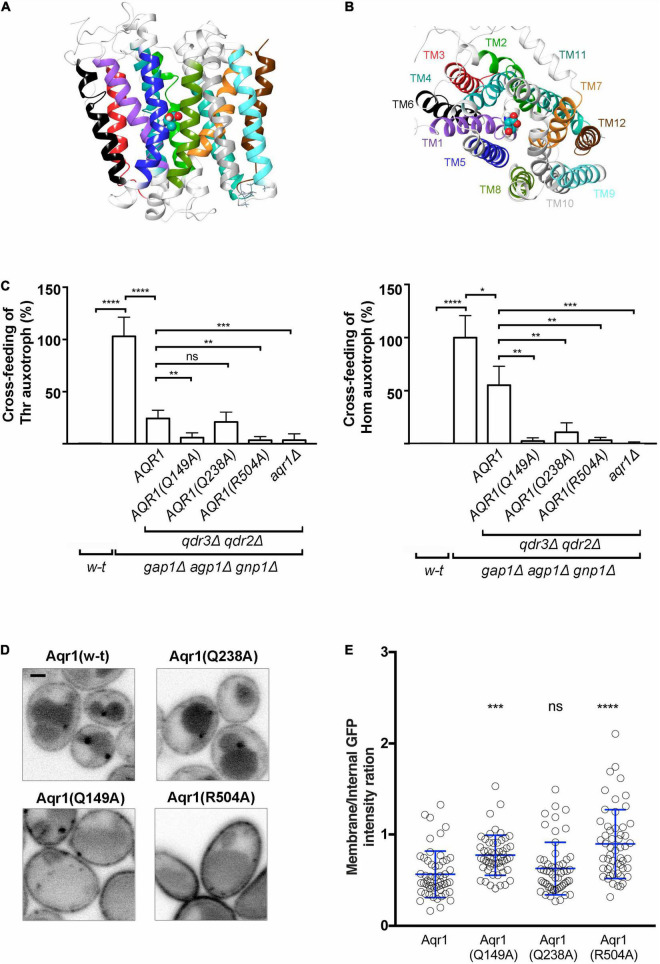
Substitutions in the predicted substrate-binding site of Aqr1 result in loss of Thr and Hom excretion. **(A,B)** Side **(A)** and top **(B)** views of the Aqr1 3D structure modeled using the MdfA 3D structure as template. The protein is depicted as a ribbon diagram with the transmembrane (TM) segments highlighted and color-coded as in Supplementary Figure 5. Threonine docked in a region delimited by using the chloramphenicol ligand in MdfA is shown as balls and sticks and colored according to the following scheme: Cyan, carbon; red, oxygen; blue, nitrogen; white, hydrogen. **(C)** The results of cross-feeding experiments illustrated in Supplementary Figure 6 were quantified as indicated in Figure 2B and normalized vs. the *gap1Δ agp1Δ gnp1Δ* strain expressing functional *AQR1*, *QDR2*, and *QDR3* genes. All strains expressed *HOM3-R* from a plasmid (pGK008). Bars represent averages of three independent experiments ± standard deviation. * indicates a statistically significant difference as determined with the unpaired *t-*test. *P < 0.0332; **P < 0.0021; ***P < 0.0002; and ****P < 0.0001. ns, not significant, *P* > 0.05. **(D,E)** Subcellular localization of w-t and mutant Aqr1 proteins C-terminally fused to GFP. **(D)** Each *AQR1-GFP* gene was expressed at its chromosomal locus under the control of its endogenous promoter. Strains were grown in minimal glucose glutamate medium up to mid-exponential phase and observed by confocal microscopy. Scale bar = 1 μm. **(E)** Quantification of the cell-surface vs. intracellular fluorescence intensity ratio ± standard deviation. *indicates a statistically significant difference as determined with the One-way ANOVA statistical test. *P < 0.0332; **P < 0.0021; ***P < 0.0002; and ****P < 0.0001. ns, not significant, *P* > 0.05.

**FIGURE 5:**
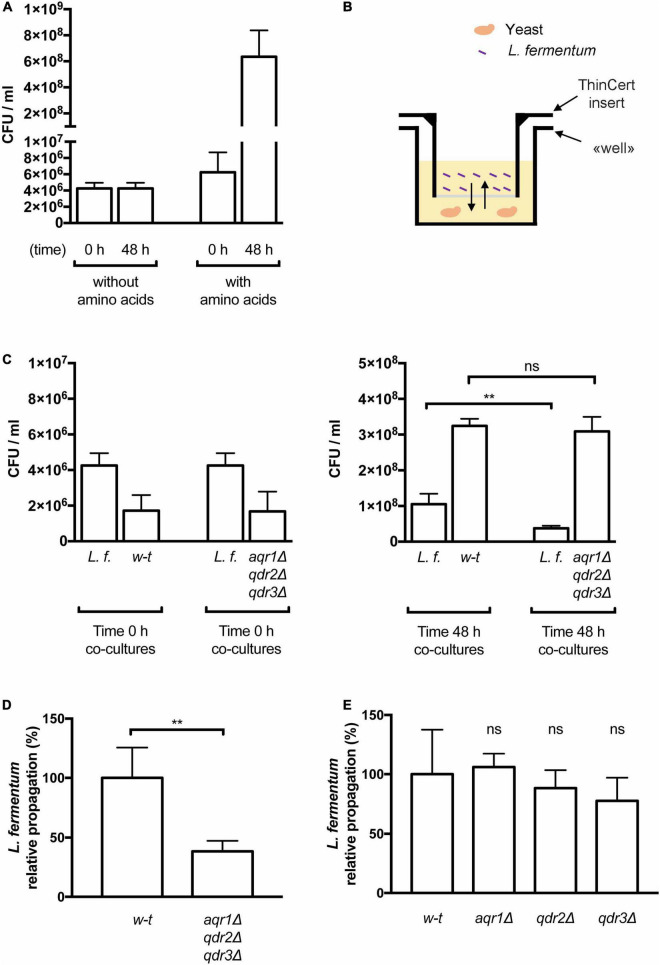
The Aqr1, Qdr2, and Qdr3 exporters promote cross-feeding of lactic acid bacteria. **(A)** Amino acid supplementation is critical for *Lactobacillus fermentum* growth. Minimal liquid 169 medium containing amino acids or not was inoculated with *L. fermentum* and growth was monitored by counting colony-forming units per milliliter (CFU/ml) just after inoculation (0 h) and after 48 h of growth. Bars represent averages of four independent experiments ± standard deviation (SD). **(B)** Schematic representation of the co-culture system. The yeast cell suspension is placed in the well and the insert prefilled with the *L. fermentum* cell suspension is placed on top of it. The arrows represent diffusion of solutes across the membrane separating the two cultures. **(C,D)** Yeast cells lacking Aqr1, Qdr2, and Qdr3 display a reduced ability to cross-feed *L. fermentum*. **(C)** Cell densities in co-cultures of *L. fermentum* (*L.f.*) with the wild-type *(w-t)* or *aqr1Δ qdr2Δ qdr3Δ* mutant yeast strain were assessed by counting CFUs just after inoculation (left) and after 48 h of growth (right). **(D)** The values presented in **(C)** were used to calculate the relative propagation of *L. fermentum* when co-cultivated with the wild-type or the *aqr1Δ qdr2Δ qdr3Δ* yeast strain. Bars represent averages of four independent experiments ± standard deviation (SD). **(E)** Single deletion of the *AQR1*, *QDR2*, or *QDR3* gene does not significantly impair cross-feeding of *L. fermentum*. Experimental conditions and calculation as in C-D, except that the yeast strains contained only an *aqr1Δ, qdr2Δ or qdr3Δ* single mutation. Bars represent averages of four independent experiments ± standard deviation. * indicates a statistically significant difference as determined with the unpaired *t-*test. **P < 0.0021. ns, not significant, *P* > 0.05.

### Microscopy

Culture samples of exponentially growing cells were laid on a thin layer of 1% agarose supplemented with glucose, vitamins and metals, and visualized at room temperature by wide-field or confocal microscopy as previously described (Gournas et al., 2017). For fluorescence quantification at the cell surface vs. internal membranes, a custom-made Fiji macro (available in the Supplementary Material) was used. In each figure, only a few cells representative of the whole population, observed in at least two independent biological replicate experiments, are shown.

### UPLC Analysis of Amino Acid Pools

Yeast cells were grown for 72 h on 165 glucose glutamate medium, collected, and extracts were prepared as previously reported (Saliba et al., 2018). The extracts were then subjected to derivatization of amino groups followed by amino acid analysis as previously described (Fayyad-Kazan et al., 2016).

### Cross-Feeding Bioassay and Imaging Analysis

Donor cells bearing plasmid pGK008 and receiver auxotrophic cells (MG734 or Σ-A3hu) were first grown on rich solid medium for 48 h. Cell samples were resuspended in water and auxotrophic cells (350 μl of 0.7 optical density at 660 nm) were spread with sterile glass beads on minimal solid medium (167) in a single large Petri dish (120 × 120 × 17mm). Then, donor cells (10 μl of 2.0 optical density at 660 nm) were dropped on top of the receiver layer to form large-size colonies. The Petri dish was incubated at 29°C for 3 days (Thr cross-feeding) or 4 days (Hom cross-feeding). To quantify growth of peripheral colonies of the auxotrophic strains, a high-resolution image of the Petri dish was first obtained by scanning. The Fiji image processing package (Schindelin et al., 2012) was then used to convert the scanned image to 8bit and adjust brightness and contrast to remove the background signal. These adjustments were identical for images corresponding to all strains analyzed in the same biological replicate. A defined square centered on each donor cell colony was then extracted from the original images. The size of the square was identical for all strains in the same biological replicate. The central colony was then covered with a black disk of the same dimensions for each donor strain. In the next step, the white colonies in the remaining area (same dimensions for each strain) were labeled using the same adjusted threshold. Finally, we used the “Analyze particles” tool with circularity values ranging from 0 to 1 (0 for no circle, 1 for perfect circle, so that all particles are measured independently to their shape) to measure the area covered by the satellite colonies around the donor cells. The values were normalized to those corresponding to colonies around the *gap1*Δ *agp1*Δ *gnp1*Δ strain displaying maximal excretion.

### Construction of the Aqr1 Models in the Inward-Facing State

A BLAST search performed on the PDB sequences (Altschul et al., 1990) identified, as appropriate templates for building Aqr1 models, the *E. coli* glycerol-3-phosphate transporter GlpT (Lemieux et al., 2005) and the *E. coli* multidrug transporter multidrug facilitator A (MdfA) complexed with chloramphenicol (Heng et al., 2015), both in the inward-facing conformation. Sequence alignments of Aqr1 with MdfA or GlpT were performed with PROMALS3D (Pei et al., 2008) and HHPRED (Söding et al., 2005). Sequence identity/similarity was calculated with the LALIGN program (Pearson, 2016), using standard parameters. Transmembrane domain predictions were performed on the Aqr1 sequence with TOPCONS (Tsirigos et al., 2015). Ten models of Aqr1 in the inward-facing occluded state were built with MODELER (Webb and Sali, 2016), five on the basis of Aqr1-GlpT sequence alignments and five others on the basis of Aqr1-MdfA sequence alignments. Docking of homoserine and threonine was performed as described previously (Ghaddar et al., 2014).

## Results

### Threonine and Homoserine Excretion Involves Aqr1, Qdr2, and Qdr3

A yeast strain expressing the dominant *HOM3-R* allele carried on a centromeric vector overproduces threonine (Thr) and its precursor homoserine (Hom) (Martin-Rendon et al., 1993). We have previously reported that when this strain additionally lacks the three main permeases catalyzing Thr and Hom uptake (Gap1, Agp1, and Gnp1), excretion of both amino acids becomes detectable. This can be visualized by spotting a dense suspension of *HOM3-R gap1*Δ *agp1*Δ *gnp1*Δ cells on a solid minimal medium onto which yeast Thr- or Hom-auxotrophic cells have been spread: after a few days of incubation, a halo of satellite colonies of auxotrophic cells develops around the large colony formed by the spotted cells (Velasco et al., 2004). In the present work, we repeated these experiments and noticed that cross-feeding of the auxotrophs was particularly pronounced on a medium containing glutamate as sole nitrogen source. This medium was therefore chosen for subsequent experiments. In experiments where intracellular Thr and Hom levels were measured by ultra performance liquid chromatography (UPLC), *HOM3-R* cells growing on this medium were found to accumulate levels 10 to 20 times as high as those found in *w-t* cells, and high levels were also observed in *HOM3-R* cells lacking the Gap1, Agp1, and Gnp1 permeases (Figure 1). In cross-feeding experiments, as expected, colonies of Thr and Hom auxotrophs formed around *HOM3-R* donor cells only when these lacked the Gap1, Agp1, and Gnp1 permeases (Figure 2A). We then used these *HOM3-R gap1*Δ *agp1*Δ *gnp1*Δ donor cells, hereafter called *3R-3P*Δ cells, to assess the contribution of the Aqr1 transporter to Thr and Hom excretion. When the *AQR1* gene was deleted in the *3R-3P*Δ strain, colonies of the auxotrophic strains still developed around the donor cells (Figure 2A). We observed, however, upon quantifying the development of these colonies in several independent experiments, that cross-feeding of both auxotrophic strains was significantly reduced. This suggests that Aqr1 contributes to excretion of both amino acids (Figure 2B).

That both Thr and Hom are still efficiently excreted when Aqr1 is not functional suggests that additional exporters are involved. Given the close phylogenetic proximity of the Qdr1-3 proteins and Aqr1 within the DHA1 protein family (Dos Santos et al., 2014), we suspected that these transporters might also contribute to Hom and Thr excretion. We thus deleted *QDR3* in the *3R-3P*Δ *aqr1*Δ strain and repeated the cross-feeding experiment. The *3R-3P*Δ *aqr1*Δ *qdr3*Δ strain largely failed to cross-feed the Hom auxotroph (Figures 2A,B), indicating that Aqr1 and Qdr3 are the main Hom-excretion proteins under the conditions of this experiment. This mutant still cross-fed the Thr auxotroph, though with reduced efficiency (Figures 2A,B), indicating that Aqr1 and Qdr3 mediate Thr excretion as well. Consistently, intracellular Hom and Thr measured by UPLC were significantly higher in *3R-3P*Δ cells lacking Aqr1 and Qdr3 (Figures 1A,B), a probable consequence of reduced excretion.

The observation that the *3R-3P*Δ *aqr1*Δ *qdr3*Δ strain still efficiently cross-feeds the Thr auxotroph suggests that one or several other proteins, besides Aqr1 and Qdr3, contribute to Thr excretion. Consistently, when the *QDR2* gene was deleted in the *3R-3P*Δ *aqr1*Δ *qdr3*Δ strain, cross-feeding of the Thr-auxotrophic cells was strongly impaired (Figure 2A,B and Supplementary Figure 1). This deletion also caused a further significant increase in intracellular Thr concentration (Figure 1B).

In conclusion, using yeast cells genetically engineered to overproduce and excrete Thr and its precursor Hom, we have shown that the Aqr1 and Qdr3 transporters mediate Hom excretion, and that these proteins together with Qdr2 promote Thr excretion. This illustrates the important role of DHA1-family transporters in amino acid excretion.

### Aqr1, Qdr2, and Qdr3 Display Significant Differences in Subcellular Localization Under Normal and Starvation Conditions

To investigate the subcellular localization of Aqr1, Qdr2, and Qdr3, we first visualized cells where each protein fused to GFP was produced from a gene placed under the control of the galactose-inducible *GAL* promoter (Figure 3). Aqr1-GFP was detected at the plasma membrane, and a substantial fraction was also found in the vacuolar lumen, as judged by colocalization with the vacuolar dye CMAC. In contrast, Qdr2-GFP and Qdr3-GFP were found mostly at the plasma membrane. The endocytosis of many yeast transporters and their sorting to the vacuole depend on their ubiquitylation *via* the essential Rsp5 ubiquitin (Ub) ligase (Lauwers et al., 2010). In the *npi1-1* strain, a viable hypomorphic mutant of *RSP5* in which many yeast transporters fail to be properly ubiquitylated (Hein et al., 1995), Aqr1-GFP localized mainly to the cell surface, like Qdr2-GFP and Qdr3-GFP (Figure 3). We also investigated the localization of Aqr1-GFP, Qdr2-GFP, and Qdr3-GFP when each was produced under the control of its own gene’s promoter at the endogenous chromosomal locus (Supplementary Figure 2). Although fluorescence signal intensity was much weaker, each protein displayed a localization pattern similar to that observed when it was overexpressed. In particular, Aqr1 differed from Qdr2 and Qdr3 in that a large fraction of the protein was detected in internal membranes and in the vacuole. We wondered if this localization pattern might be due to altered trafficking of the protein caused by its fusion to GFP, but the Aqr1-GFP construct is functional, as judged by its ability to promote excretion of Hom and Thr in a *HOM3-R* strain lacking Qdr2 and Qdr3 (Supplementary Figure 3). Further analysis of Aqr1-GFP localization in mutants altered at specific intracellular trafficking steps (Supplementary Figure 4 and supportive text) revealed that at least part of the protein cycles constantly between the plasma and internal membranes, in keeping with previous observations (Velasco et al., 2004).

We next sought to determine whether localization of the Aqr1, Qdr2, and Qdr3 exporters might be altered in starved cells. We first examined cells after 90 min of nitrogen starvation (Figure 3). This treatment caused total relocation of Aqr1-GFP from the cell surface to the vacuole. A fraction of the Qdr3-GFP was also targeted to the vacuole, whereas Qdr2-GFP remained at the cell surface. In the *npi1-1* strain, both Aqr1-GFP and Qdr3-GFP remained at the cell surface. We also analyzed the localization of each exporter after shifting cells to a glucose-free medium (Figure 3). This condition is known to cause rapid Ub-dependent down-regulation of many plasma-membrane transporters, a response contributing to cell survival under these conditions (Lang et al., 2014). In wild-type cells shifted to glucose starvation for 90 min, Aqr1-GFP and Qdr3-GFP were efficiently targeted to the vacuole. This down-regulation was reduced though still effective in the *npi1-1* mutant. In contrast, Qdr2-GFP remained at the plasma membrane (Figure 3).

In conclusion, although Aqr1, Qdr2, and Qdr3 share high sequence similarity and are all three involved in amino acid export, they differ appreciably as regards their turnover at the plasma membrane under nutrient sufficiency and starvation.

### Substitutions in the Predicted Substrate-Binding Site of Aqr1 Result in Loss of Thr and Hom Excretion

Solving at the atomic level the structures of Aqr1, Qdr2, and Qdr3 would be most helpful toward understanding how these proteins function as amino acid exporters. As such experimental data are unavailable, we adopted a structural modeling approach focusing on Aqr1, chosen as a representative DHA1-family amino acid exporter.

After comparing the Aqr1 sequence with those of proteins available in the Protein Data Bank (PDB), we selected the crystal structures of two *Escherichia coli* proteins as templates for building Aqr1 structural models: the MdfA multidrug-resistance protein (Multidrug Facilitator A; PDB code: 4ZOW) (Heng et al., 2015) and the GlpT glycerol-3-phophate antiporter (PDB code: 1PW4) (Lemieux et al., 2005), both in the Inward Facing (IF) conformation. These bacterial transporters belong to the MFS superfamily and their transport activity is driven by the proton gradient at the plasma membrane (Edgar and Bibi, 1997; Lemieux et al., 2005). For each bacterial transporter template, five 3D models of Aqr1 were built and their stereochemical quality was verified (see details in Supplementary Figure 5). One of these Aqr1 structural models is shown in Figures 4A,B.

MdfA has been crystallized in a complex with chloramphenicol, one of its substrates (Heng et al., 2015). Using the 3D models of Aqr1 superimposed onto the MdfA structure template, we were able to predict that the substrate-binding pocket of Aqr1 is mainly shaped by residues in TM1, -2, -4, -5, -6, -7, and -11. To identify, among these residues, those potentially involved in forming substrate contacts, we performed docking of Thr and Hom in the predicted binding pocket of Aqr1. In a significant number of poses predicted by the models, a small number of Aqr1 residues were repeatedly found to form hydrogen bonds or salt bridges with both Thr and Hom (Gln 149, Gln 238) or with Hom only (Arg 504) (Supplementary Figures 5A,B and Supplementary Table 1). These residues of Aqr1 are thus predicted to be directly involved in recognition of the excreted amino acids.

To assess this prediction, we used CRISPR-Cas9 to introduce nucleotide substitutions into the chromosomal *AQR1* gene of the *3R-3P*Δ *qdr2*Δ *qdr3*Δ strain (Mans et al., 2015), where Aqr1 is the sole functional Thr/Hom exporter. The resulting strains expressing the Aqr1(Q149A), Aqr1(Q238A), or Aqr1(R504A) variant were then used as donors in Thr and Hom cross-feeding experiments (Figure 4C and Supplementary Figure 6). Excretion of both amino acids was found to be markedly reduced in cells expressing Aqr1(Q149A) or Aqr1(R504A), whereas cells expressing Aqr1(Q238A) displayed significant reduction of Hom excretion only. We also measured intracellular concentrations of Thr and Hom in the mutant strains (Supplementary Figure 7). In agreement with the results of the cross-feeding experiments, cells expressing the mutant Aqr1 variants displayed higher accumulation of both amino acids, most likely due to their impaired excretion. To assess the possibility that reduced Thr and Hom excretion in the mutant strains might be due to mislocalization of the Aqr1 variants, we analyzed cells in which the wild-type and mutant chromosomal *AQR1* genes were fused in frame to *GFP*. Initial analysis showed the mutant forms of Aqr1-GFP to localize roughly like the wild-type protein, to the cell surface, internal puncta, and the vacuole (Figure 4D). Quantification of the fluorescence signals further revealed more abundant Aqr1(R504A)-GFP and Aqr1(Q149A)-GFP at the cell surface than in internal membranes (Figure 4E). This suggests that these Aqr1 mutants, which are the less active in Thr and Hom excretion (Figure 4C), are also less prone to endocytosis and turnover.

In conclusion, these results support the predictions based on Aqr1 structural modeling and show that residues R504, Q149, and Q238 in the substrate-binding cavity of Aqr1 play an important role in amino acid excretion. The results are also consistent with the view that endocytic downregulation of Aqr1 is promoted by its transport activity, as observed for several amino acid transporters (Ghaddar et al., 2014; Gournas et al., 2017).

### The Aqr1, Qdr2, and Qdr3 Exporters Promote Cross-Feeding of a Lactic Acid Bacterium

Yeast and lactic acid bacteria (LAB) interact metabolically during natural fermentation of several foods and beverages (D’Souza et al., 2018). Previous works have shown that yeast can supply LAB with several amino acids which the latter cannot synthesize naturally (Challinor and Rose, 1954; Ponomarova et al., 2017). In addition to mediating excretion of Thr and Hom, Aqr1, Qdr2, and Qdr3 might export additional amino acids and thus contribute to cross-feeding LAB. To assess this possibility, we first adjusted the composition of the liquid minimal buffered medium used in the above experiments to make it compatible with growth of *Lactobacillus fermentum*. Specifically, citrate used as a buffer and found to impede growth of this bacterium was replaced with MES hydrate, and additional vitamins were provided (see section “Materials and Methods”). The results presented in Figure 5A show that *L. fermentum* grew well on this medium only when supplied with amino acids, confirming that this LAB species is auxotrophic for several amino acids (Deguchi and Morishita, 1992; Oliva Neto and Yokoya, 1997). We then cultivated cells in wells consisting of two compartments separated by a solute-permeable membrane (Figure 5B). One compartment was inoculated with yeast cells of the wild-type or the *aqr1*Δ *qdr2*Δ *qdr3*Δ mutant strain, and the other with *L. fermentum*. Just after inoculation and after 2 days of growth, the cell density was quantified in culture samples by counting the number of colony-forming units (CFU/ml). The results first revealed that *L. fermentum*, when co-cultivated with wild-type yeast, propagated rather well (Figure 5C), though less than the yeast cells. This shows that the bacterium was cross-fed with amino acids supplied by the yeast. Importantly, the relative propagation of *L. fermentum* vs. yeast was reduced _∼_2.5-fold when the bacterium was co-cultivated with the *aqr1*Δ *qdr2*Δ *qdr3*Δ yeast mutant strain (Figures 5C,D). This reduced cross-feeding was not observed when *L. fermentum* was co-cultivated with an *aqr1*Δ, *qdr2*Δ, or *qdr3*Δ single mutant (Figure 5E). These results indicate that Aqr1, Qdr2, and Qdr3 contribute redundantly to the excretion of amino acids promoting growth of *L. fermentum*.

## Discussion

It has been known for decades that yeast can excrete amino acids (Challinor and Rose, 1954; Grenson, 1973). More recent studies have shown that this leakage is not due to cell lysis but is rather a natural process favored by nitrogen overflow (Paczia et al., 2012; Ponomarova et al., 2017). The yeast proteins catalyzing excretion of amino acids have been less studied than those mediating amino acid uptake from the environment or sequestration within organelles. More generally, the yeast transporters involved in metabolite leakage remain poorly characterized, likely because they are harder to track genetically and to characterize functionally than those catalyzing nutrient uptake (Van Belle and André, 2001). The main conclusion of our study is that the closely related Aqr1, Qdr2, and Qdr3 transporters of yeast contribute actively to excretion of amino acids. Furthermore, they most likely play a direct role therein. In support of this view, structural modeling of Aqr1 and identification, by docking, of residues predicted to mediate recognition of Thr and Hom have enabled us to design and isolate Aqr1 substitution mutants that are indeed unable to excrete these amino acids, a phenotype not due to their improper targeting to the plasma membrane.

As DHA1-family members localizing to the plasma membrane, Aqr1 and Qdr2/3 likely function as amino-acid/H^+^-antiporters exploiting the H^+^ gradient established by the Pma1 H^+^-pump. Under nitrogen or carbon starvation, we observe efficient downregulation of Aqr1 and Qdr3 *via* Ub-dependent endocytosis and targeting to the vacuole. This downregulation, by limiting amino acid leakage, might in principle be advantageous under nutrient limitation, but as downregulation under starvation has been observed for many plasma-membrane transporters involved in nutrient uptake (Kahlhofer et al., 2021), it might be a general response allowing cells to retrieve free amino acids from permease degradation (Jones et al., 2012). Furthermore, Qdr2 was not downregulated under the starvation conditions tested, although we cannot rule out that its fusion to GFP might have prevented its endocytosis artifactually. The Aqr1 exporter differs from Qdr2 and Qdr3 in that it displays a higher Ub-dependent turnover at the plasma membrane. Furthermore, this turnover is impeded by activity-loss-causing substitutions in the Aqr1 substrate-binding pocket. This observation raises the interesting possibility that Aqr1 ubiquitylation and endocytosis might be accelerated upon substrate transport, as illustrated for a growing number of amino acid permeases (Ghaddar et al., 2014; Gournas et al., 2017; Kahlhofer et al., 2021).

Our initial evidence that Aqr1 and Qdr2/3 play a role in amino acid export was obtained with cells overproducing and exporting Thr and Hom. In these cells, Hom excretion is impaired when both Aqr1 and Qdr3 are lost, and further inactivation of Qdr2 is needed to prevent Thr excretion. Hence, functional redundancy exists between these three proteins. Importantly, excretion of Thr and Hom was detectable only if their uptake was impaired by inactivation the Gap1, Agp1, and Gnp1 permeases. The exact reason for this constraint is not fully understood. The simplest interpretation is that overproduced Thr and Hom are excreted and then rapidly re-assimilated by the same cells *via* the three amino acid permeases. This first scenario amounts to a futile cycle, but as both amino acids were artificially overproduced, such an excretion-reuptake process might occur despite its energy cost. Alternatively, excretion and reuptake might be achieved by different cell subpopulations coexisting within the colony that cross-fed the surrounding auxotrophs. Yeast colonies have indeed been reported to contain several cell types displaying different metabolic states, although such differentiation has mostly been observed under carbon limitation (Váchová and Palková, 2018; Varahan et al., 2019). According to this scenario, the Thr and Hom released by one cell subpopulation in the colony would diffuse through the solid medium and cross-feed the auxotrophs more readily when the permeases promoting their reuptake by another cell subpopulation are not active.

The other experimental setup used in this study to illustrate the roles of Aqr1 and Qdr2/3 in amino acid excretion is growth for 2 days in a liquid glucose medium adapted for co-cultivation of *Lactobacillus fermentum*. In these experiments we found wild-type cells to excrete enough amino acids to sustain growth of the auxotrophic bacteria used, in keeping with observations of another study (Ponomarova et al., 2017). Our results further show that concomitant loss of Aqr1 and Qdr2/3 in yeast donor cells causes a reduction of *L. fermentum* growth. This phenotype is not observed when only one of the three proteins is lacking, which again illustrates functional redundancy between them. In this cross-feeding system, excretion of amino acids was detectable even though the Gap1, Agp1, and Gnp1 permeases, capable of assimilating a very wide range of amino acids, were functional. A possible explanation is that the genes encoding these permeases were less expressed. For example, transcription of both *GAP1* and *AGP1* are subject to nitrogen catabolite repression (Jauniaux and Grenson, 1990; Abdel-Sater et al., 2004), which should indeed be operational under the conditions of the experiment.

As members of the DHA1 family, yeast Aqr1 and Qdr2/3 are similar in sequence to bacterial and fungal multidrug resistance proteins (Sá-Correia et al., 2009). Furthermore, they were initially described as proteins conferring resistance to toxic concentrations of multiple compounds, including short-chain monocarboxylic acids, the antimalarial drug quinidine, the herbicide barban, and the anticancer agents cisplatin and bleomycin (Tenreiro et al., 2002, 2005; Vargas et al., 2004, 2007). To reconcile these observations with our finding that these proteins function as amino acid exporters, one might point out that the Aqr/Qdr proteins are broad-spectrum exporters, potentially involved in excretion of other metabolites besides amino acids. This view raises questions, however: what is the actual physiological role of these proteins and under which conditions do they function? One possibility is that these exporters are low-affinity systems which can excrete many different compounds only when these are present in the cell at relatively high concentrations. For instance, these proteins could be particularly useful under metabolic overflow conditions. Although such leakage would come with an inevitable price, namely occasional export of useful metabolites, it could be compensated by reuptake *via* high-affinity import systems. Alternatively, the main role of the Aqr/Qdr proteins could actually be to excrete amino acids. This model is also conceivable, because the proposed role of Aqr1, Qdr2, or Qdr3 in multidrug resistance was based mostly on growth assays showing that cells lacking one of these proteins are more sensitive to certain drugs, whereas cells overexpressing them display the opposite phenotype. These phenotypes might be indirect consequences of metabolic perturbations associated with altered amino acid export. This view fits with the observation that the DHA1-family protein Dtr1, responsible for export of the natural compound bisformyl-dityrosine (the major building block of the ascospore surface), also confers increased resistance to several organic acids and drugs when overexpressed (Felder et al., 2002). Other DHA1-family members, however, most likely play a direct role in resistance to multiple drugs. Accordingly, their corresponding genes are under the control of transcription factors mediating pleiotropic drug resistance (do Valle Matta et al., 2001). This contrasts with *DTR1*, expressed only during meiosis (Felder et al., 2002), and with the *AQR1*, *QDR2*, and *QDR3* genes, regulated by nitrogen or amino acid availability (Sá-Correia et al., 2009).

Finally, although our results show that Aqr1 and Qdr2/3 contribute importantly to cross-feeding LAB, a mutant strain lacking all three is still capable of supporting limited LAB growth. Further work is thus needed to characterize the amino acid exporters responsible for this residual amino acid excretion. More generally, much investigation is needed to present a full picture of the yeast nutrient exporters involved in mutualistic interactions with other microorganisms and to characterize their biochemical properties and the conditions under which they are most active.

## Data Availability Statement

The original contributions presented in the study are included in the article/Supplementary Material, further inquiries can be directed to the corresponding author/s.

## Author Contributions

GK, CG, MP, IG, and BA: conceptualization. GK and MP: data production. GK, CG, MP, IG, and BA: data curation and validation. MP, IG, and BA: project supervision. GK, MP, IG, and BA: writing of draft version. CG, MP, IG, and BA: writing review. IG and BA: project administration. All authors contributed to the article and approved the submitted version.

## Conflict of Interest

Patent PCT/EP2021/060851 of ULB describes yeast mutant strains for reducing contamination of lactic acid bacteria in yeast cultures. The authors declare that the research was conducted in the absence of any commercial or financial relationships that could be construed as a potential conflict of interest.

## Publisher’s Note

All claims expressed in this article are solely those of the authors and do not necessarily represent those of their affiliated organizations, or those of the publisher, the editors and the reviewers. Any product that may be evaluated in this article, or claim that may be made by its manufacturer, is not guaranteed or endorsed by the publisher.
