# Supplementary material for: Overlapping Roles of Yeast Transporters Aqr1, Qdr2, and Qdr3 in Amino Acid Excretion and Cross-Feeding of Lactic Acid Bacteria
Source: Front Microbiol. 2021 Nov 23;12:752742. doi: 10.3389/fmicb.2021.752742 (PMC8649695; doi:10.3389/fmicb.2021.752742)
Supplement: Supplementary file 1 [file Data_Sheet_1.zip › FINAL SUPPLEMENTARY PROOFS.pdf]

Overlapping roles of yeast transporters Aqr1, Qdr2, and Qdr3 in amino acid excretion and cross-feeding of lactic acid bacteria

George C. Kapetanakis, Christos Gournas, Martine Prévost,
Isabelle Georis, Bruno André

Supplementary Figures and Tables

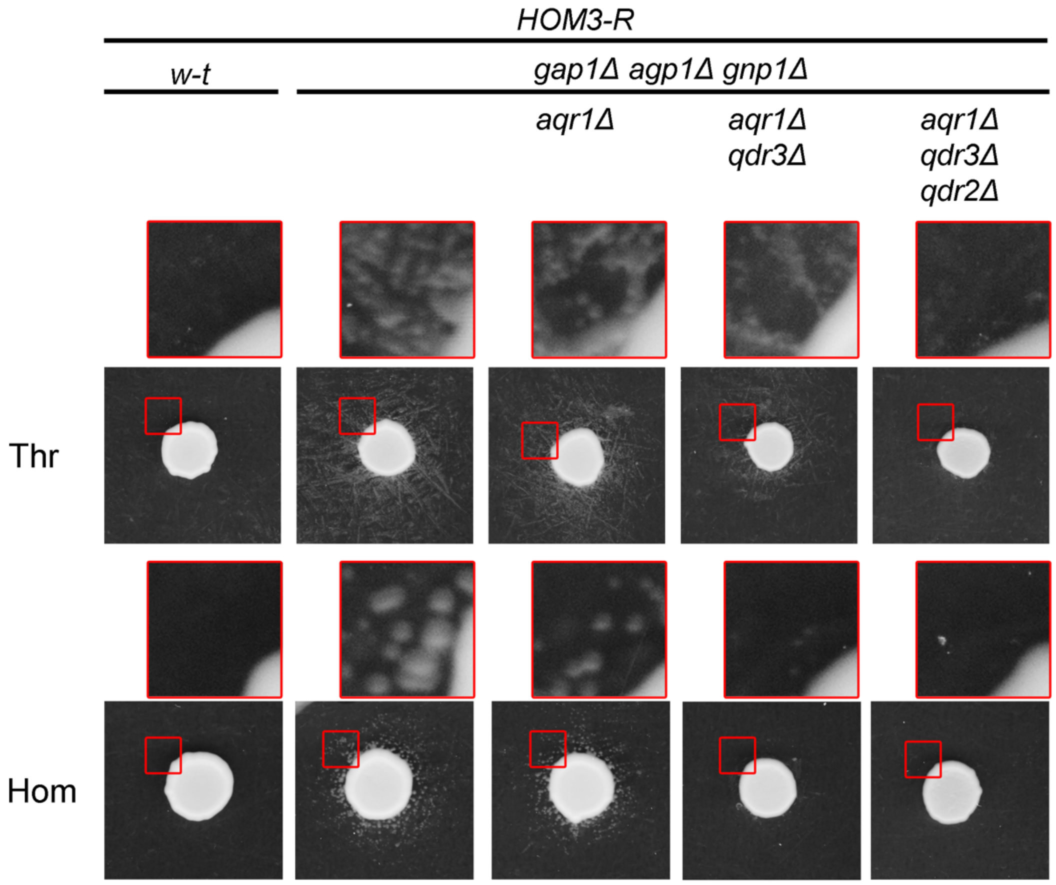

Supplementary Figure 1. Results of cross-feeding experiments illustrated in Figure 2A with original scanned images.

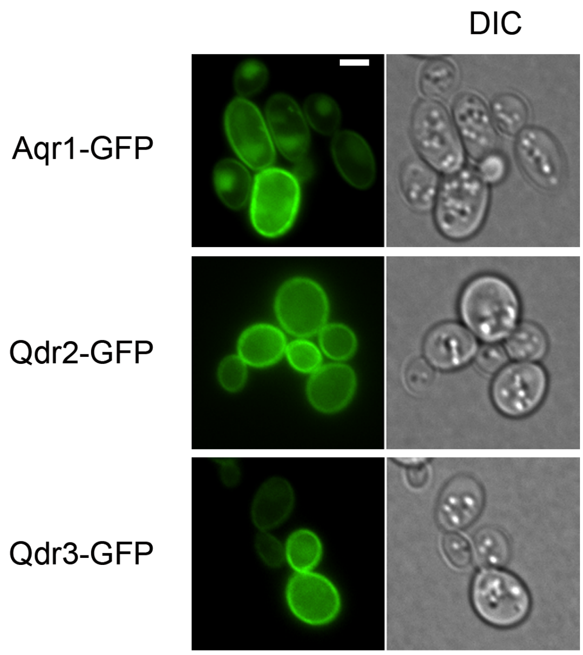

Supplementary Figure 2. Subcellular localization of Aqr1, Qdr2, and Qdr3 under native expression conditions. Wild-type cells expressing an *AQR1-GFP* (FV905), *QDR3-GFP* (FV843), or *QDR2-GFP* (FV951) hybrid gene at its chromosomal locus from its native promoter were grown exponentially in minimal glucose medium containing glutamate as sole nitrogen source and examined by epifluorescence microscopy. Scale bar is 2 μm.

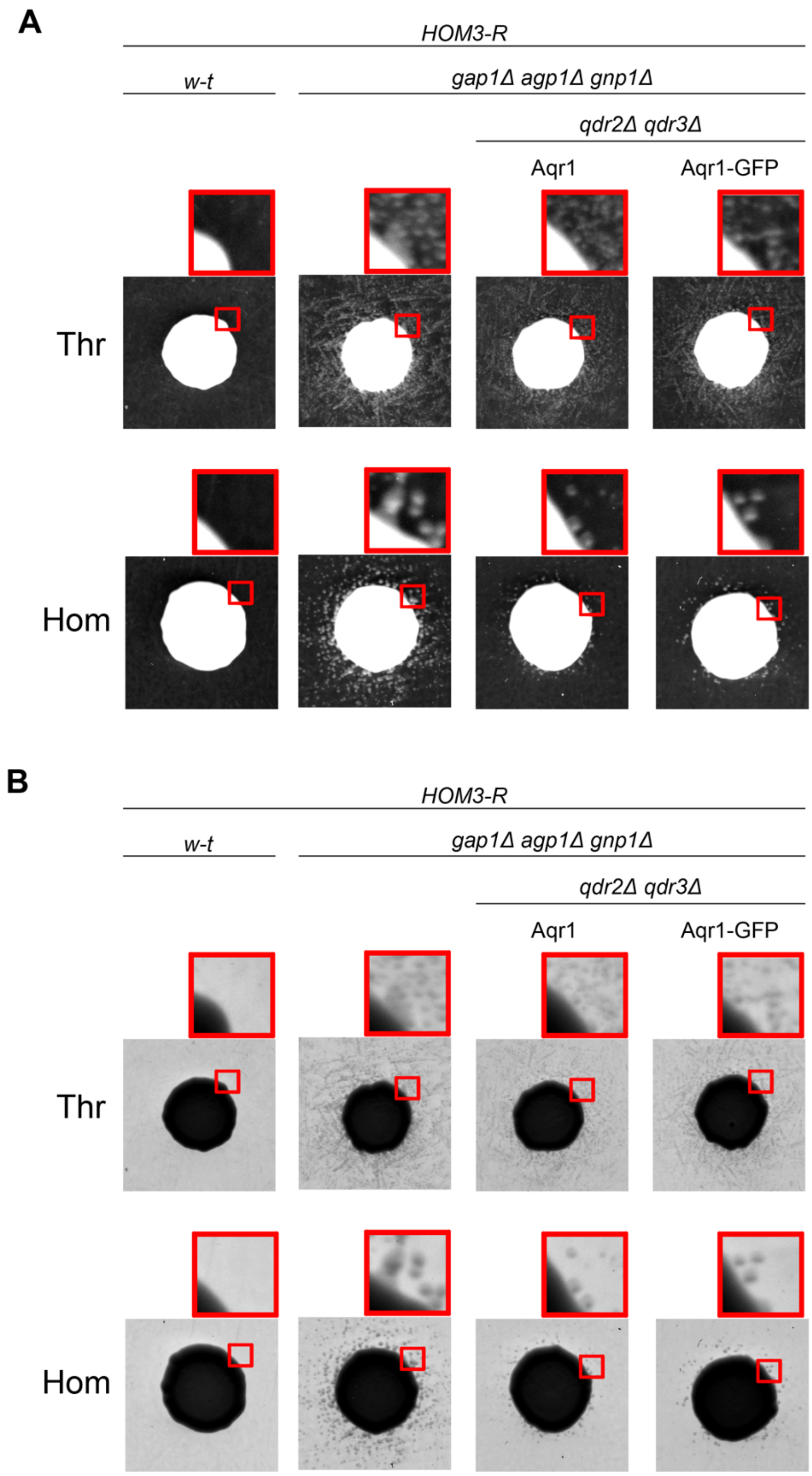

Supplementary Figure 3. Cross-feeding experiment showing that Aqr1 fused to GFP is functional. Cell suspensions prepared from wild-type and mutant strains (genotypes indicated), all transformed with the *HOM3-R*-containing plasmid (pGK008), were dropped over a layer of yeast threonine (Thr) or homoserine (Hom) auxotrophic mutants to form a large colony. Upper images in red squares correspond to

enlargements of a zone at the periphery of the central donor colony. The cells were incubated for 3-4 days at 29oC. Glutamate was the sole nitrogen source in the medium. Strains used: 23344c (*w-t*), JA248 (*gap1Δ gnp1Δ agp1Δ*), GK068 (*gap1Δ gnp1Δ agp1Δ qdr2Δ qdr3Δ*), GK103 (*gap1Δ gnp1Δ agp1Δ qdr2Δ qdr3Δ* AQR1-ECFP), MG734 (*thr4Δ*), and Σ-A3hu (*hom3Δ*). (A) Images corresponding to the original scans obtained for a representative experiment. (B) Negative of the image shown in A.

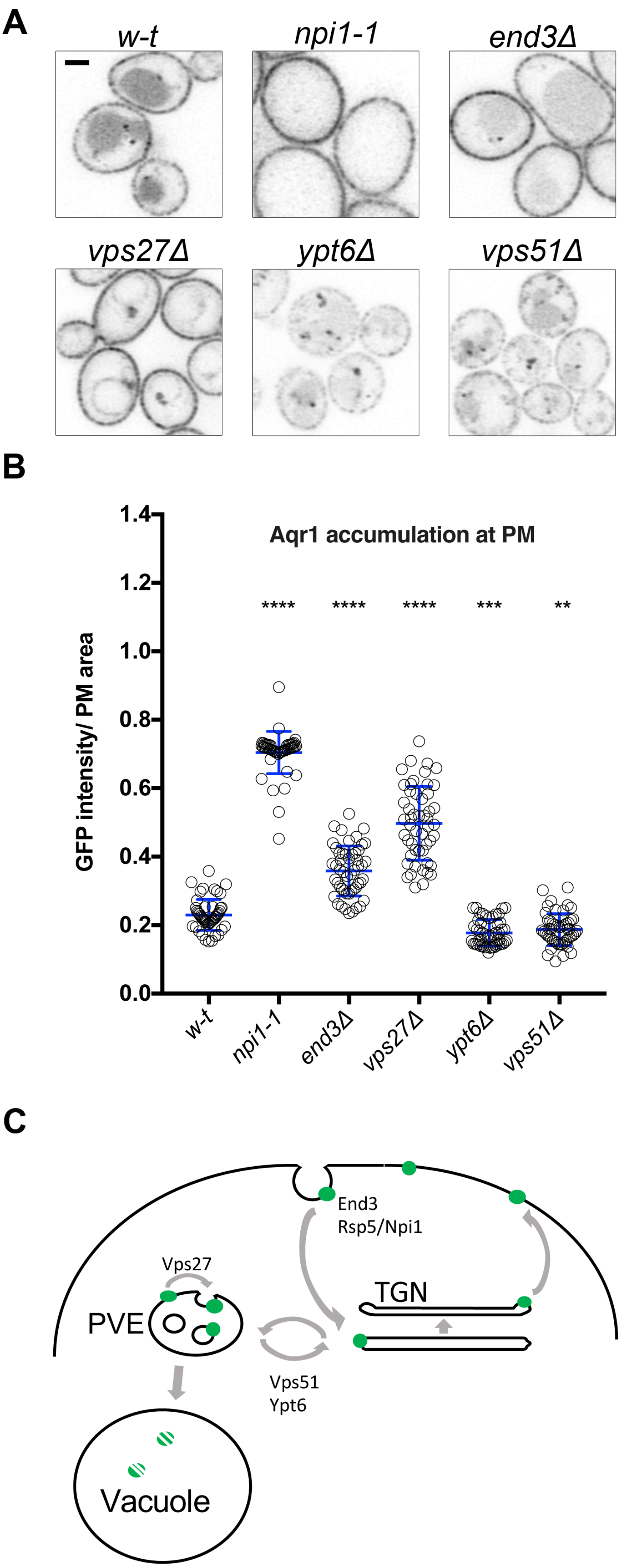
A
w-t
npi1-1
end3Δ
vps27Δ
ypt6Δ
vps51Δ
B
Aqr1 accumulation at PM
GFP intensity/ PM area
1.4
1.2
1.0
0.8
0.6
0.4
0.2
0.0
****
****
****
***
**
w-t
npi1-1
end3Δ
vps27Δ
ypt6Δ
vps51Δ
C
End3
Rsp5/Npi1
Vps27
PVE
TGN
Vps51
Ypt6
Vacuole

Supplementary Figure 4. Intracellular traffic of Aqr1. (A) Subcellular localization of Aqr1-GFP. Cells of strains GK001 (*AQR1*-G*FP w-t*), JA618 (*AQR1-GFP npi1-1*), JA629 (*AQR1-GFP end3Δ*), JA625 (*AQR1-GFP vps27Δ*), GK099 (*AQR1-GFP ypt6Δ*), and GK101 (*AQR1-GFP vps51Δ*) were grown exponentially in minimal glucose glutamate medium and examined by confocal microscopy. (B) Quantification of cell-surface Aqr1-GFP abundance. Strains as in A. The fluorescence intensity normalized vs. surface area was measured with ImageJ. *indicates a statistically significant difference as determined with the one-way ANOVA statistical test. ** P<0.0021; *** P<0.0002; **** P<0.0001. (C) A model of Aqr1 intracellular traffic. A fraction of the Aqr1 located at the plasma membrane is constantly internalized by endocytosis. Once at the Golgi, part of the Aqr1 is recycled to the cell surface via the *trans*-Golgi network (TGN). Another part traffics to the Pre-Vacuolar Endosomal compartment (PVE), where it is sorted via the Multi Vesicular Body (MVB) pathway to intra-endosomal vesicles. This allows subsequent delivery of Aqr1 to the vacuole, where it is degraded (see text below for more details).

Supportive text associated with Supplementary Figure 4. Aqr1 differs from Qdr2 and Qdr3 in that it is distributed between the plasma and internal membranes. To further investigate this subcellular distribution, we used high-resolution confocal microscopy to locate Aqr1-GFP in wild-type and several mutants impaired in specific steps of intracellular trafficking (Supplementary Figure 4A). In wild-type cells, Aqr1-GFP produced under the control of its own promoter was found at the plasma membrane, in multiple intracellular puncta, and in the vacuole. In the *end3Δ* mutant, where endocytosis of many plasma-membrane proteins is impaired (Raths et al., 1993; Weinberg and Drubin, 2012), Aqr1-GFP was mostly present at the cell surface. Furthermore, quantification of the fluorescence signal normalized vs. surface area revealed a significantly higher density of Aqr1-GFP at the plasma membrane in this strain (Supplementary Figure 4B). These phenotypes were even more pronounced in the *npi1-1* strain, a hypomorphic mutant affected in the gene encoding the Rsp5 ubiquitin-ligase and in which the endocytosis of many proteins is impaired (Hein et al., 1995; Weinberg and Drubin, 2012). Staining by Aqr1-GFP of the vacuole was indeed undetectable, and still higher amounts of Aqr1-GFP accumulated at the plasma membrane (Supplementary Figures 4A, B). Hence, newly synthesized Aqr1 first traffics to the plasma membrane, where part of the protein undergoes ubiquitin-

dependent endocytosis and targeting to the vacuole. According to recent studies, internalized proteins first reach the *trans*-Golgi Network (TGN), from where they can either be recycled to the plasma membrane or delivered to the vacuole (Becuwe and Léon, 2014; Day et al., 2018). Targeting of these proteins to the vacuole first requires their transit from the TGN to the Pre Vacuolar-Endosome (PVE), where they are sorted into internal vesicles typically forming Multi Vesicular Bodies (MVB). The final step is fusion of the PVE/MVB with the limiting membrane of the vacuole, leading to degradation of the proteins in the vacuolar lumen (Becuwe and Léon, 2014; Day et al., 2018). Among the multiple factors required for protein sorting into MVB vesicles is Vsp27, a component of the ESCRT-0 complex (Bilodeau et al., 2003). In *vps27Δ* cells, Aqr1-GFP largely failed to be delivered to the vacuole and accumulated, rather, in punctate structures likely corresponding to enlarged PVEs (class E compartments), typically observed in this mutant. *vps27Δ* cells also displayed a stronger Aqr1-GFP signal at the cell surface, indicating that the protein having failed to be sorted into the MVB pathway tends to recycle to the cell surface. Furthermore, a faint Aqr1-GFP signal was located at the peripheral membrane of the vacuole. Such missorting phenotypes are typically observed when MVB sorting is defective. We finally examined Aqr1-FP in two other mutants. These strains lack Ypt6, the yeast Rab6 homolog, or Vps51, a component of the Golgi-associated VFT/GARP complex, both required for retrograde endosome-to-Golgi trafficking (Siniossoglou et al., 2000; Conibear et al., 2003; Reggiori et al., 2003). It has been reported that a lack of either of these proteins also impacts internalized transporters, which fail to colocalize with TGN and rather accumulate in puncta (Becuwe and Léon, 2014). In the *ypt6Δ* and *vps51Δ* mutants, Aqr1-GFP similarly accumulated in many small puncta. Furthermore, the intensity of the Aqr1-GFP signal at the cell surface was significantly reduced (Fig. S4B). This suggests that a limited fraction of Aqr1 that normally traffics through the TGN and PVE tends to recycle from these compartments to the cell surface.

In conclusion, these results show that a fraction of the Aqr1 reaching the plasma membrane is actively sorted into endocytic vesicles in a ubiquitin-dependent manner. Once internalized, part of the protein is sorted from the Golgi to the vacuole via the PVE compartment and MVB pathway, whereas another seems to be actively recycled to the plasma membrane.

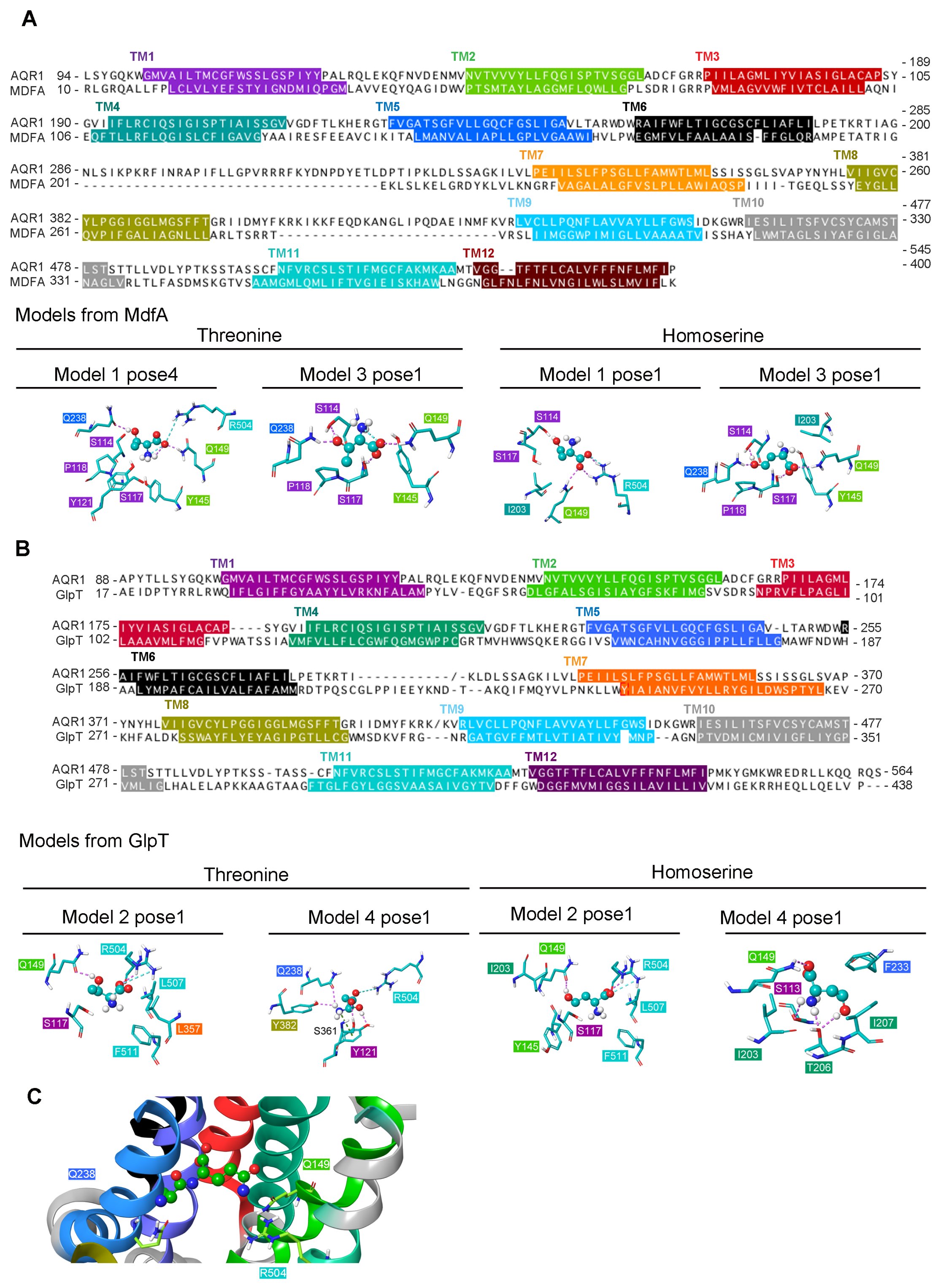

Supplementary Figure 5. Structural modeling of Aqr1. (A-B) Top. Sequence alignment of Aqr1 with MdfA (A) or GlpT (B). The TM helices, as determined by PDBTM on the crystal structures of MdfA or GlpT and as predicted by the TOPCONS tool on the Aqr1 sequence, are highlighted in different colors. Bottom. Close-up views of representative docking poses of threonine and homoserine in Aqr1 3D structures

modeled with either the MdfA (A) or the GlpT (B) crystal structure as template (see Table S1 for more details). Thr and Hom are shown as ball-and-stick representations. The surrounding residues are represented by sticks and their label is framed using the color code of the TM to which the residue belongs. Residues are colored according to the following scheme. Cyan, carbon; red, oxygen; blue, nitrogen; white, hydrogen. Hydrogen bonds and ionic interactions are shown as pink and light blue broken lines, respectively. (C) The substrate-binding pocket of the PepTSt H^+-coupled oligopeptide transporter bound to the Ala-Gly dipeptide (PDB code: 5OXK) (Martinez Molledo et al., 2018) was superposed on the equivalent region in the Aqr1 3D model. Residues Gln149, Gln238, and Arg504 in Aqr1 appear in the direct environment of the Ala-Gln dideptide, further validating the Aqr1 3D models and docking data.

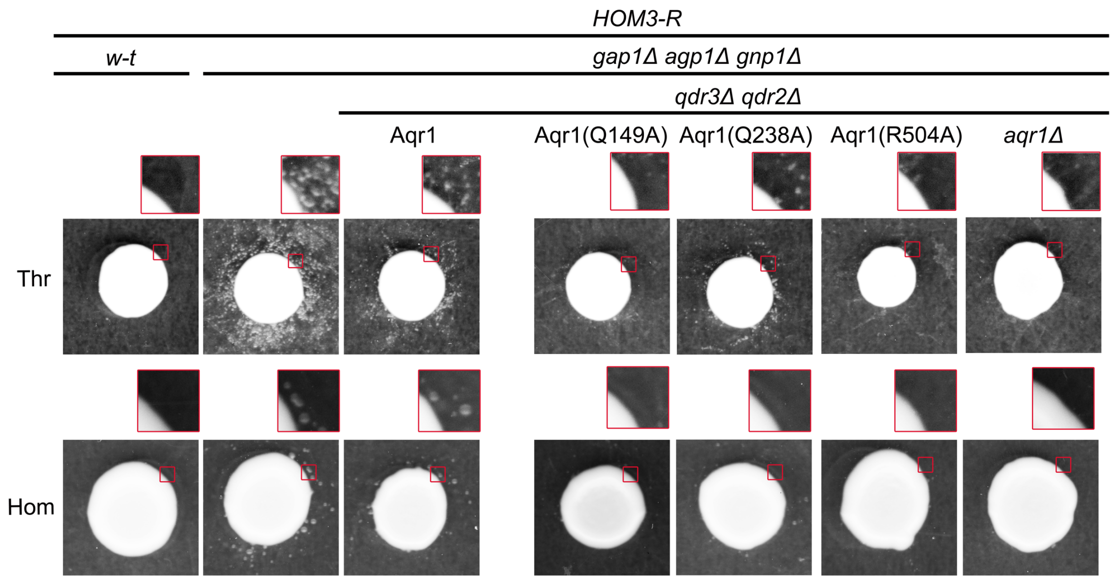

Supplementary Figure 6. Aqr1 mutants with substitutions in the predicted substrate-binding site are inactive. Cross-feeding, on solid medium, of threonine (Thr) and homoserine (Hom) auxotrophic mutants surrounding large colonies of the wild-type (*w-t*) and mutant strains (genotypes indicated) expressing *HOM3-R* from a plasmid (pGK08). Cells were grown on a minimal glucose medium with glutamate as sole nitrogen source. The images show the results obtained in one representative experiment. Strains used: 23344c (*w-t*), GK068 (*gap1Δ gnp1Δ agp1Δ qdr3Δ qdr2Δ*), GSD05 (*gap1Δ agp1Δ gnp1Δ qdr3Δ qdr2Δ* AQR1(Q149A)) GSD01 (*gap1Δ agp1Δ gnp1Δ qdr3Δ qdr2Δ* AQR1(Q238A)), GSD03 (*gap1Δ agp1Δ gnp1Δ qdr3Δ qdr2Δ* AQR1(R504A)), JA248 (*gap1Δ gnp1Δ agp1Δ*), GK085 (*gap1Δ gnp1Δ agp1Δ aqr1Δ qdr3Δ qdr2Δ*), MG734 (*thr4Δ*), and Σ-A3hu (*hom3Δ*).

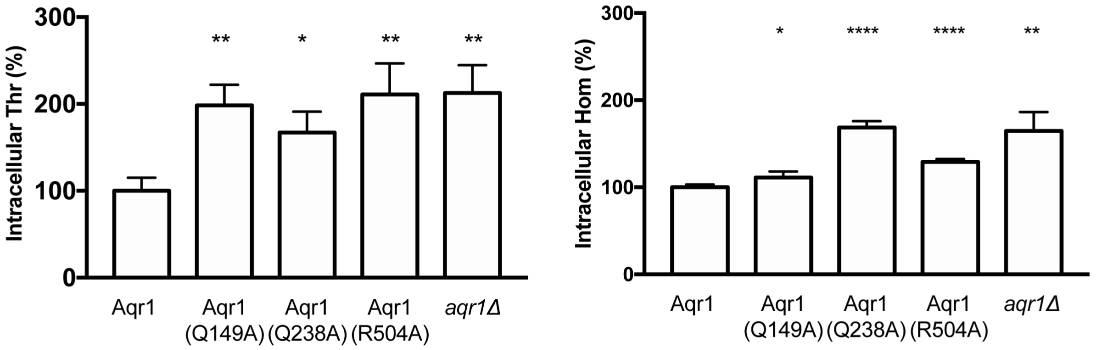

Supplementary Figure 7. Substitutions impairing Aqr1 function cause a significant increase in overproduced threonine and homoserine. Intracellular concentrations of threonine (Thr) (A) and homoserine (Hom) (B) were quantified by UPLC in cells grown in minimal glucose glutamate medium for 3 days. The values were normalized vs. those measured in the strain expressing wild-type Aqr1. All strains expressed *HOM3-R* from a plasmid (pGK007). Bars represent the averages of three independent experiments ± standard deviation. * indicates a statistically significant difference as determined with the unpaired *t-test*. * P<0.0332; ** P<0.0021; **** P<0.0001. Strains used: GK068 (*gap1Δ gnp1Δ agp1Δ qdr3Δ qdr2Δ*), GSD05 (*gap1Δ agp1Δ gnp1Δ qdr3Δ qdr2Δ AQR1(Q149A)*), GSD03 (*gap1Δ agp1Δ gnp1Δ qdr3Δ qdr2Δ AQR1(R504A)*), GSD01 (*gap1Δ agp1Δ gnp1Δ qdr3Δ qdr2Δ AQR1(Q238A)*), and GK085 (*gap1Δ gnp1Δ agp1Δ aqr1Δ qdr3Δ qdr2Δ*).

Supplementary Table 1. Docking poses and scores of threonine and homoserine binding in 10 Aqr1 3D models produced with the MdfA or the GlpT protein as template.

AQR1--MdfA		**Threonine**		**Homoserine**	
		**Interacting residues (H bonds and/or salt bridges)**	**Docking score (kcal/mol)**	**Interacting residues (H bonds and/or salt bridges)**	**Docking score (kcal/mol)**
**MODEL 1**	**pose1**	GLN238, TYR121	-2,515	GLN149, AGR504	-6.359
	**pose2**	GLN238, TYR121	-2,030	--	
	**pose3**	GLN238	-1,67	--	
	**pose4**	GLN238, GLN149, ARG504	1,403	--	
**MODEL 2**	**pose1**	TYR121	-0,377	GLN149	-2.795
	**pose2**	--		GLN149	-2.264
**MODEL 3**	**pose1**	GLN 149, GLN238, SER114, SER117	-3.490	GLN 149, GLN238, SER114, SER117	-3.650
	**pose2**	GLN238, SER114, SER117	-3.354	GLN238, GLN 149, SER117	-2.575
	**pose3**	--		GLN238, GLN 149, SER117	-2.515
	**pose4**	--		SER117	-2.489
**MODEL 4**	**pose1**	GLN238	-2,945	GLN 149, GLN238, SER114	-0.262
	**pose2**	GLN238	-2,056	GLN 149, GLN238, SER114	-0.042
	**pose3**	--		GLN238, SER113	-0.012
**MODEL 5**	**pose1**	THR 206	-4,175	GLN 149, THR206, GLY110	-2.986
	**pose2**	THR 206, GLY 110	-3,737	--	
	**pose3**	THR206	-3,728	--	
	**pose4**	THR 206	-3,696	--	

AQR1--GlpT		Threonine		Homoserine	
		**Residues interacting (H bonds and salt bridges)**	**Docking score (kcal/mol)**	**Residues interacting (H bonds and salt bridges)**	**Docking score (kcal/mol)**
**MODEL 1**	**pose1**	GLN 238	-4,079	GLN 238, SER471	-4.037
	**pose2**	GLN 238	-3	--	
**MODEL 2**	**pose1**	GLN 149, ARG 504	-3.534	GLN 149, ARG 504	-3.188
	**pose2**	SER 117	-2.938	SER 117	-2.877
	**pose3**	SER 117, GLN 149, ARG 504	-2.543	--	
	**pose4**	SER 117	-1,589	--	
**MODEL 3**	**pose1**	TYR382, SER117	-3,283	TYR145, TYR382, SER360	-0,494
	**pose2**	TYR382, SER117	-2,895	TYR 121, SER 117	-0.377
	**pose3**	--		SER117	-0.327
	**pose4**	--		SER117, TYR145, TYR382, SER360	0.481
	**pose5**	--		SER117, TYR121, TY382	3.527
**MODEL 4**	**pose1**	TYR121, GLN238, TYR382, SER361	-3,047	THR206, GLN 149	-2.242
	**pose2**	GLY 110, SER113	-2,682	TYR382, ARG504, GLN238, TYR121, PHE511	-1.736
	**pose3**	GLN149, THR 206	0,68	TYR382, ARG504, GLN238, TYR121, PHE511	-1.635
	**pose4**			TYR382, ARG504, GLN238, TYR121, PHE511	0.054
**MODEL 5**	**pose1**	GLN149, SER508	-3,178	SER 508, GLN 149	-2.839
	**pose2**	--		GLN 149, GLN238	-1.616
	**pose3**	--		SER 114, PHE233, THR206	2.379

Supplementary Table 2: Strains used in this study.

code	genotype	ref.
23344c	*ura3*	Laboratory collection
FV905	*AQR1-yECitrine ura3*	this study
FV843	*QDR3-yECitrine ura3*	this study
FV951	*QDR2-yECitrine ura3*	this study
FV847	$P_{GAL1\text{-}10}$*-AQR1- yECitrine ura3*	this study
FV849	$P_{GAL1\text{-}10}$*- QDR3- yECitrine ura3*	this study
FV1013	$P_{GAL1\text{-}10}$*- QDR2-yECitrine ura3*	this study
35870a	$P_{GAL1\text{-}10}$*-AQR1-yECitrine npi1-1 ura3*	this study
35864a	$P_{GAL1\text{-}10}$*- QDR3-yECitrine npi1-1 ura3*	this study
35873b	$P_{GAL1\text{-}10}$*- QDR2-yECitrine npi1-1 ura3*	this study
GK001	*AQR1-GFP ura3*	this study
GK099	*AQR1-GFP ypt6Δ ura3*	this study
GK101	*AQR1-GFP vps51Δ ura3*	this study
JA618	*AQR1-GFP npi1-1 ura3*	(Velasco et al., 2004)
JA625	*AQR1-GFP vps27Δ ura3*	this study
JA629	*AQR1-GFP end3Δ ura3*	this study
JA248	*gap1Δ gnp1Δ agp1Δ ura3*	(Velasco et al., 2004)
GK065	*gap1Δ gnp1Δ agp1Δ aqr1Δ ura3*	this study
GK082	*gap1Δ gnp1Δ agp1Δ aqr1Δ qdr3Δ ura3*	this study
GK085	*gap1Δ gnp1Δ agp1Δ aqr1Δ qdr3Δ qdr2Δ ura3*	this study
GK068	*gap1Δ gnp1Δ agp1Δ qdr3Δ qdr2Δ ura3*	this study
GSD01	*gap1Δ agp1Δ gnp1Δ qdr3Δ qdr2Δ* Aqr1(Q238A) *ura3*	this study
GSD03	*gap1Δ agp1Δ gnp1Δ qdr3Δ qdr2Δ* Aqr1(R504A) *ura3*	this study
GSD05	*gap1Δ agp1Δ gnp1Δ qdr3Δ qdr2Δ* Aqr1(Q149A) *ura3*	this study
GK103	*gap1Δ gnp1Δ agp1Δ qdr3Δ qdr2Δ* Aqr1-ECFP *ura3*	this study
GK104	*gap1Δ agp1Δ gnp1Δ qdr3Δ* qdr2Δ Aqr1(Q238A)-ECFP *ura3*	this study
GK105	*gap1Δ agp1Δ gnp1Δ qdr3Δ qdr2Δ* Aqr1(R504A)-ECFP *ura3*	this study
GK106	*gap1Δ agp1Δ gnp1Δ qdr3Δ qdr2Δ* Aqr1(Q149A)-ECFP *ura3*	this study
GK121	*aqr1Δ qdr3Δ qdr2Δ ura3*	this study
MG734	*thr4Δ*	Laboratory collection
Σ-A3hu	*hom3Δ ura3*	Laboratory collection

Supplementary Table 3: Plasmids used in this study.

code	Plasmid description	ref.
pFL038	YCp-*URA3*	Laboratory collection
pGK007	YCpHom3(Gly452Asp), *URA3*	this study
pGK008	YCpHom3(Glu282Asp), *URA3*	this study

REFERENCES

Becuwe, M., and Léon, S. (2014). Integrated control of transporter endocytosis and recycling by the arrestin-related protein Rod1 and the ubiquitin ligase Rsp5. *Elife* 3:e03307. doi: 10.7554/eLife.03307.

Bilodeau, P.S., Winistorfer, S.C., Kearney, W.R., Robertson, A.D., and Piper, R.C. (2003). Vps27-Hse1 and ESCRT-I complexes cooperate to increase efficiency of sorting ubiquitinated proteins at the endosome. *J Cell Biol* 163(2)**,** 237-243. doi: 10.1083/jcb.200305007.

Conibear, E., Cleck, J.N., and Stevens, T.H. (2003). Vps51p mediates the association of the GARP (Vps52/53/54) complex with the late Golgi t-SNARE Tlg1p. *Mol Biol Cell* 14(4)**,** 1610-1623. doi: 10.1091/mbc.e02-10-0654.

Day, K.J., Casler, J.C., and Glick, B.S. (2018). Budding Yeast Has a Minimal Endomembrane System. *Dev Cell* 44(1)**,** 56-72.e54. doi: 10.1016/j.devcel.2017.12.014.

Hein, C., Springael, J.Y., Volland, C., Haguenauer-Tsapis, R., and André, B. (1995). NPl1, an essential yeast gene involved in induced degradation of Gap1 and Fur4 permeases, encodes the Rsp5 ubiquitin-protein ligase. *Mol Microbiol* 18(1)**,** 77-87. doi: 10.1111/j.1365-2958.1995.mmi_18010077.x.

Martinez Molledo, M., Quistgaard, E.M., Flayhan, A., Pieprzyk, J., and Löw, C. (2018). Multispecific Substrate Recognition in a Proton-Dependent Oligopeptide Transporter. *Structure* 26(3)**,** 467-476.e464. doi: 10.1016/j.str.2018.01.005.

Raths, S., Rohrer, J., Crausaz, F., and Riezman, H. (1993). end3 and end4: two mutants defective in receptor-mediated and fluid-phase endocytosis in Saccharomyces cerevisiae. *J Cell Biol* 120(1)**,** 55-65. doi: 10.1083/jcb.120.1.55.

Reggiori, F., Wang, C.W., Stromhaug, P.E., Shintani, T., and Klionsky, D.J. (2003). Vps51 is part of the yeast Vps fifty-three tethering complex essential for retrograde traffic from the early endosome and Cvt vesicle completion. *J Biol Chem* 278(7)**,** 5009-5020. doi: 10.1074/jbc.M210436200.

Siniossoglou, S., Peak-Chew, S.Y., and Pelham, H.R. (2000). Ric1p and Rgp1p form a complex that catalyses nucleotide exchange on Ypt6p. *Embo j* 19(18)**,** 4885-4894. doi: 10.1093/emboj/19.18.4885.

Velasco, I., Tenreiro, S., Calderon, I.L., and Andre, B. (2004). Saccharomyces cerevisiae Aqr1 is an internal-membrane transporter involved in excretion of amino acids. *Eukaryot Cell* 3(6)**,** 1492-1503. doi: 10.1128/ec.3.6.1492-1503.2004.

Weinberg, J., and Drubin, D.G. (2012). Clathrin-mediated endocytosis in budding yeast. *Trends Cell Biol* 22(1)**,** 1-13. doi: 10.1016/j.tcb.2011.09.001.
